# Cutting Efficiency of Diamond Grinders on Composite and Zirconia

**DOI:** 10.3390/ma17112596

**Published:** 2024-05-28

**Authors:** Martin Rosentritt, Thomas Strasser, Maerit-Martha Mueller, Michael Benno Schmidt

**Affiliations:** Department of Prosthetic Dentistry, UKR University Hospital Regensburg, 93042 Regensburg, Germany

**Keywords:** diamond, grinder, composite, zirconia, cutting efficiency

## Abstract

This in vitro study was carried out to compare the cutting efficiency of diamond grinders on zirconia and resin-based composite materials. Grinders were employed with a special holder for the handpiece to apply a constant load (160 g) for resin-based composite (8 cuts, 40 s each) and zirconia materials (4 cuts, 5 min each; n = 10 for each material and grinder). To assess the efficiency of the grinders, weight measurements of the material were taken before and after the grinding process. Scanning electron micrographs were captured for instrument surfaces before and after testing and for the resulting surface of the materials. In the resin-based composite group, there were significant differences in weight removal between the burs for both the baseline (first cut; *p* = 0.009) and removal after the eighth cut (*p* = 0.049). Statistically significant decreases in weight removal compared to the baseline values were noted for the third, fourth, sixth, and seventh steps (*p* ≤ 0.046). For the zirconia group, significant differences existed in weight removal between the burs for the baseline (first cut; *p* < 0.001) and removal after the fourth cut (*p* < 0.001). A significant positive correlation was observed between removal and the number of cuts (Pearson: 0.673; *p* < 0.001). A statistically significant decrease in removal compared to the respective baseline value was found for the fourth step (*p* = 0.006). The initial wear removal and durability significantly differed between the grinders used on resin-based composite and zirconia. Achieving comparable weight removal took five times longer when grinding zirconia compared to the resin-based composite.

## 1. Introduction

Resin-based composite and zirconia are common and widespread as computer-aided design (CAD) and computer-aided manufacturing (CAM) materials for dental applications. With their different material properties, they represent the extreme upper and lower limits of hardness (210 HV vs. 380 HV), modulus (15 GPa vs. 210 GPa), and strength (330 MPa vs. 1200 MPa) of tooth-colored materials and therefore place different demands on handling and grinding [1,2,3,4,5,6,7].

These materials are customarily processed with diamond grinders in clinical practice, predominantly to shape the form or to adjust the occlusion [8]. The diamonds are furthermore used to remove, adjust, or polish prosthetic restorations. Extra- or super-coarse (125–180 µm), coarse (100–150 µm), and regular (75–125 µm) diamonds are used for significant material reduction, whereas fine (20–40 µm), super-fine (10–30 µm), and ultra-fine (4–14 µm) diamonds are applied for grinding and polishing [9]. In dental practice the grinders are distinguished between ultra-fine (white, M 4–M 14; 8 µm), extra-fine (yellow, M 10–M 36; 25 µm), fine (red, M 27–D 76; 46 µm), medium (blue, D 64–D 126; 107 µm), coarse (green, D 107–D 181; 151 µm), and very coarse (black, D 151–D 213; 181 µm).

The properties of diamond grinders are determined by certain specifications: They are offered with a high variation in the amount, type, shape, and size of the diamonds, resulting in different cutting efficiencies and lifespans of the bur. Burs should sustain optimal cutting efficiency throughout the treatment. Various metrics can be employed to assess cutting efficiency, for example, changes in the weight of the test substrate over a certain time, volumetric cutting rate, and depth of cut over a fixed time [10,11,12,13].

These parameters depend on the design and fabrication of the grinders and can therefore vary greatly. Newer studies with a similar experimental set up to ours showed that a single patient bur is more effective than a multi-patient bur and that diamond burs are more effective for cutting zirconia than tungsten burs [14,15].

Therefore, the purpose of the present study was to compare the cutting efficiency of diamond grinders on zirconia and resin-based composite CAD/CAM materials. The hypothesis of this study was that different types of diamond grinders with different diameters do not show different removal capacity on the materials.

## 2. Materials and Methods

**Figure 1:**
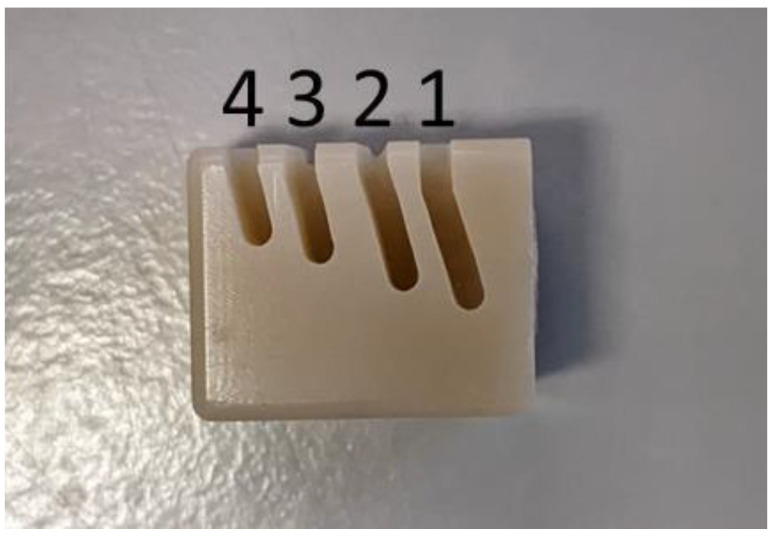
Exemplary grinding steps 1–4 on resin-based composite.

Specimens of zirconia or resin-based composite were ground with different grinders. The resulting weight removal was determined by measuring the difference in weight (MXX-612, Denver Instrument, Behemia, NY, USA) of the specimens before and after the grinding process. A special holder for the handpiece was developed to ensure a reproducible load (160 g) and grinding process. Due to the different material properties and significantly different removal efficiency, the time and number of grinding steps varied between the two materials. Pretests on the individual materials were performed to determine material dependent grinding time and number of grinding steps. Four cuts were made per side of each blank (Figure 1). Because a grinder lasts significantly longer and is more effective during use on resin-based composite, 8 cuts, 40 seconds each were used for the resin-based material and 4 cuts, 5 min. each for zirconia. All values for the weight removal were statistically compared to the weight removal after the first cut (baseline). Ten specimens were investigated for every material and grinder system. Statistical analysis was performed with one-way ANOVA, Bonferroni, and Pearson comparison (α = 0.05, SPSS, Chicago, IL, USA).

For resin-based composite (Grandio blocs 14L A3.5 HT; VOCO, D), instruments (Z881-016C-FG “ABACUS”; 881-016C-FG; 881-014TC-FG “TURBO”; 881-014C-FG; NTI-Kahla, D; 6881.314.016; 6881.314.014; Komet Dental, D) were used in 8 steps for 40 seconds per step (n = 10, Table 1). Resin-based composite blanks were used as delivered. 

For 3Y-TZP zirconia (Cercon base 30 colored; Dentsply, D), instruments (K881-016M-FG “Z-CUT”; 881-016M-FG; all NTI-Kahla, 881.314.016; Komet Dental, D) were used in 4 steps for 5 minutes per step (n = 10, Table 1). 3Y-TZP blanks were cut into cubes (12 mm × 14 mm × 18 mm) and sintered (1350 °C; Cercon heat, Dentsply, D) before testing. 

## 3. Results

### 3.1. Resin-Based Composite

**Figure 2:**
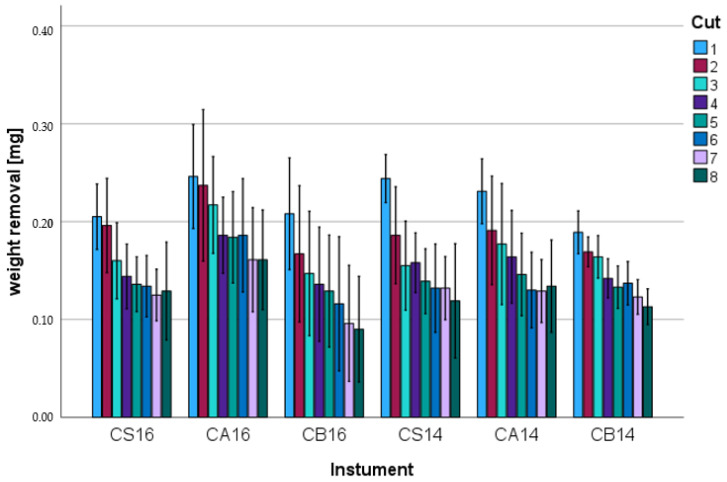
Weight loss [mg] for resin-based composite.

The measured weight removal for the resin-based composite varied between 0.25 ± 0.05 g (CS16; first step) and 0.09 ± 0.05 g (CB16; eight step). For all instruments, a continuous decrease in weight removal was observed as the number of cuts increased. The baseline removal (first cut; *p* = 0.009) and the removal after the eight cut (*p* = 0.049) were significantly different between the burs. The removal and number of the cuts showed no correlation (Pearson: −0.27, *p* = 0.333). A statistically significant (*p* ≤ 0.046) decrease in the weight removal compared to the respective baseline value was shown for the third step (CS16; CS14), fourth step (CA14; CB14), sixth step (CB16) and seventh step (CA16). Figure 2 and Table 2 show the weight removal after the individual cuts. The different diameters in identical groups showed no significant differences (*p* = 0.069) for lower weight reduction.

### 3.2. Zirconia

**Figure 3:**
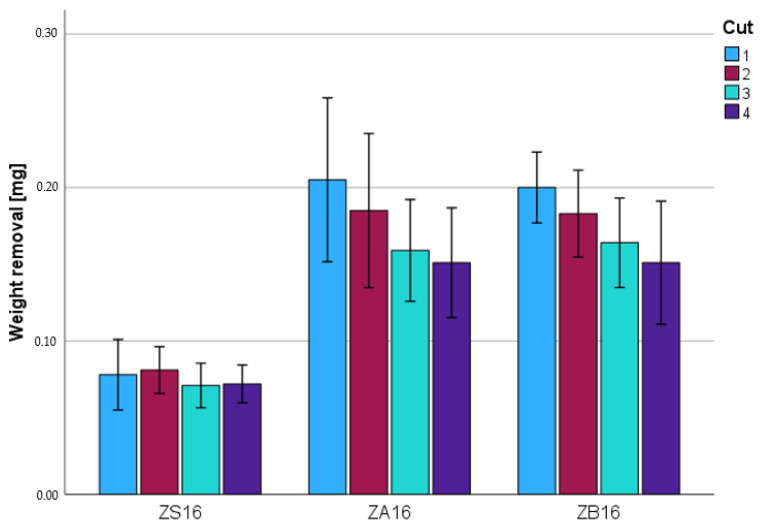
Weight loss [mg] for zirconia.

The weight removal for zirconia varied between 0.21 ± 0.05 g (ZA16; first step) and 0.07 ± 0.01 g (ZS16; third step). For both standard instruments, a continuous decrease in weight removal was observed as the number of cuts increased. The baseline removal (first cut; *p* < 0.001) and the removal after the fourth cut (*p* < 0.001) were significantly different between the burs. The removal and number of cuts showed a significant correlation (Pearson: 0.673; *p* < 0.001). A statistically significant (*p* = 0.006) decrease in the removal compared to the respective baseline value was shown for the fourth step (ZB16). The instruments (ZS16, ZA16) did not show a statistically significant decrease in removal (*p* = 1.000 for ZS16) compared to the baseline value (*p* ≥ 0.056 for ZA16). Figure 3 and Table 3 show the weight removal after the individual cuts.

**Figure 4:**
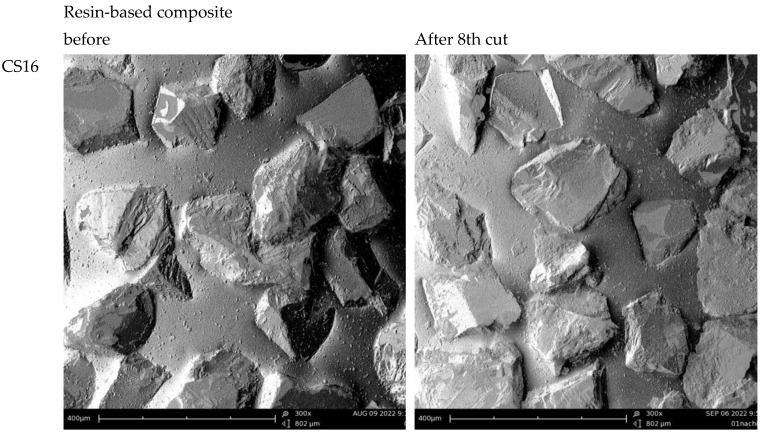

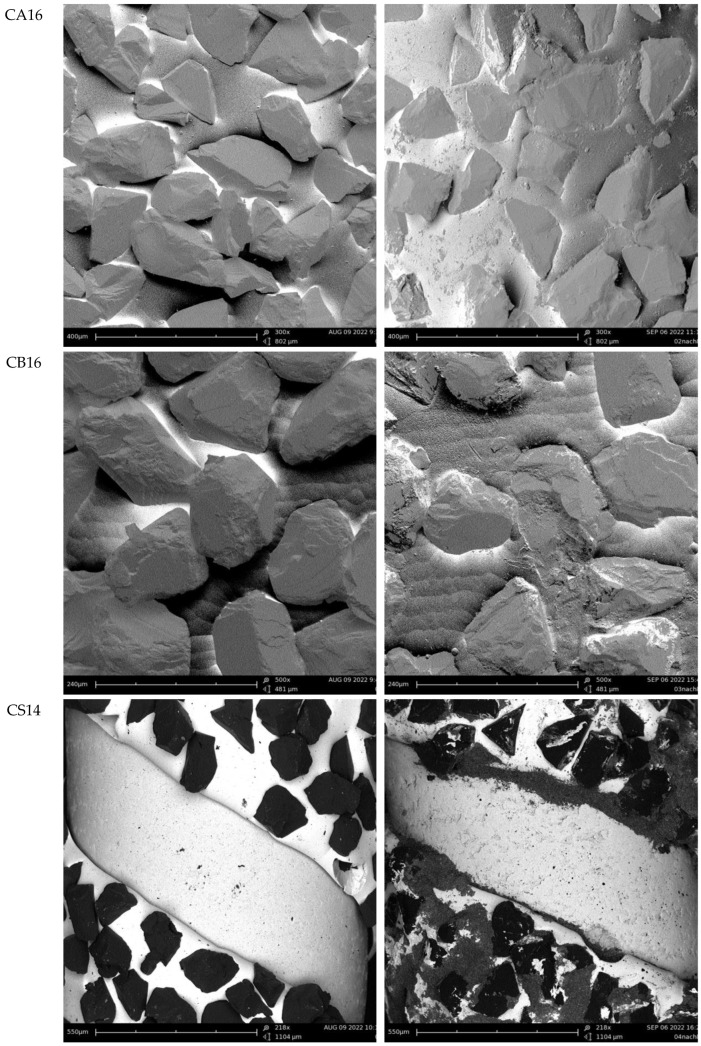

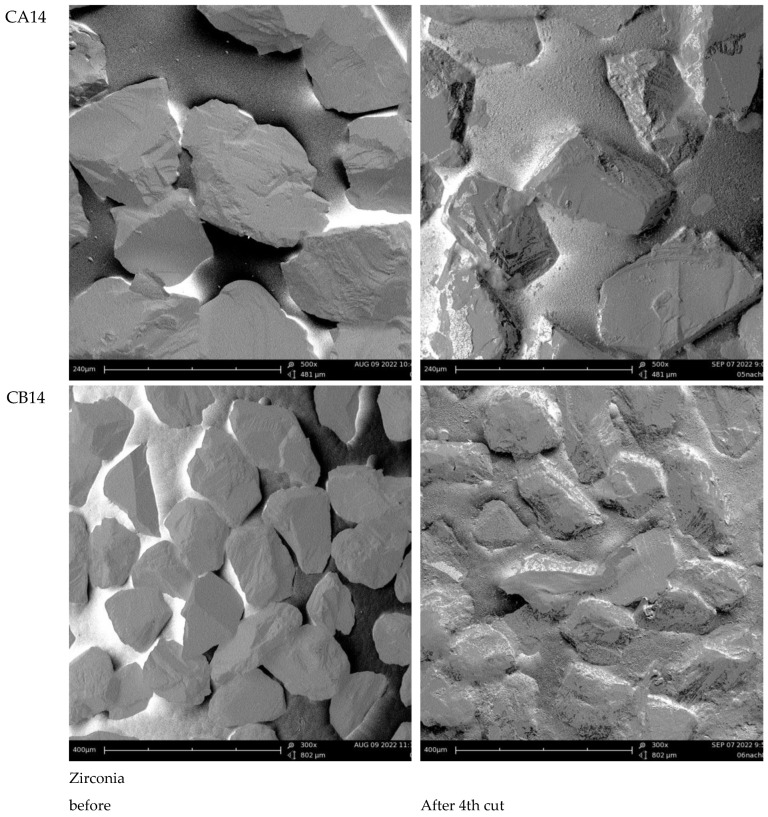

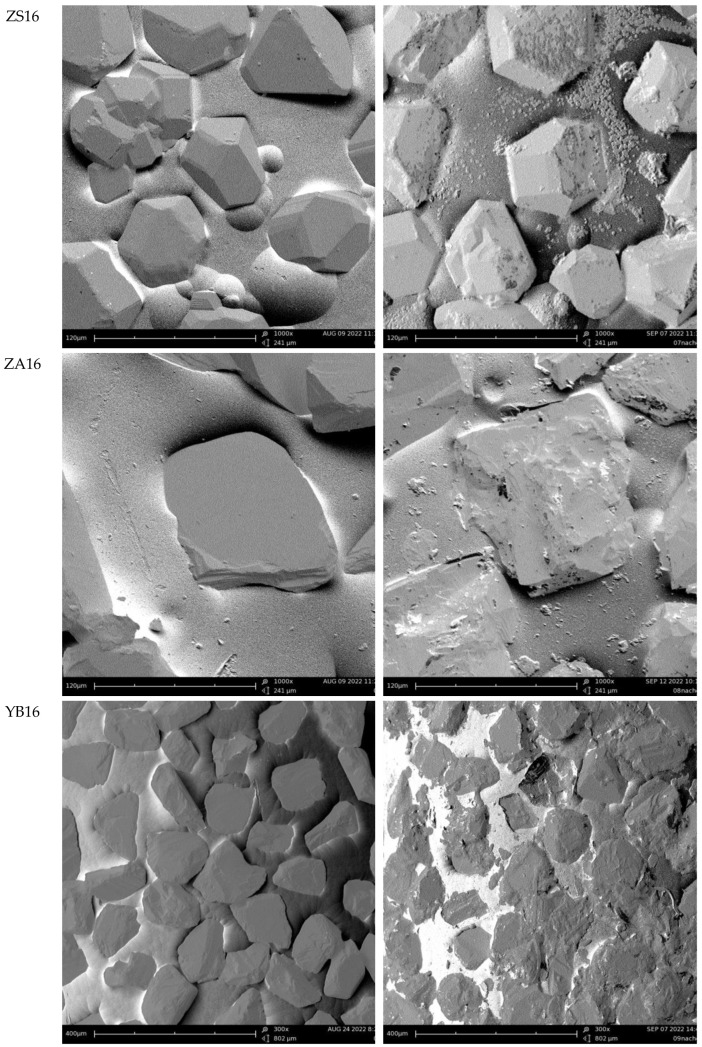
SEM images of the grinder surface before and after use.

The scanning electron microscopy (SEM) images showed that the reduced weight removal was primarily due to wear of the diamonds. Rounding of the profile of the individual diamond grains and wear and loss of material along the contours were observed. Only in the case of the ZB16 instrument was a complete loss of diamond particles observed. For the diamonds of instrument ZS16, no superficial wear traces or damage were seen in the SEM images (Figure 4).

## 4. Discussion

The hypothesis of this study that different types of diamond grinders do not show different removal capacity on the materials considered here could not be confirmed. The weight reduction of the investigated instruments varied significantly between the different grinder systems. The influence of the grinder diameter (1.4 mm vs. 1.6 mm) on the processing of the composite could not be confirmed.

### 4.1. Resin-Based Composite

The systems examined differed significantly in their initial weight removal for the resin-based composite. The maximal weight reduction was between 0.19 and 0.25 mg for the different diamond systems after the first cut. None of the grinders exceeded eight cuts without a reduction in cutting performance. Similar behavior has already been observed for resin-based composite treatment [16].

However, clear differences could be found: A significant reduction in weight removal, towards the initial weight removal at the first cutting run, was achieved between three and seven cuts. The conventional grinders showed slight advantages over the special grinders. Conventional grinders with larger diameters only showed advantages in the initial cut. One conventional system showed the highest weight results even after eight cuts. The systems examined differed significantly in their final weight removal after eight cuts. For the 1.6 mm grinders, the special coating of the diamonds did not provide any advantages. For the 1.4 mm grinders, the special turbo grinder provided slight advantages in terms of the initial removal but was at the same level as the conventional grinders in the final removal stage. Further studies should be carried out to determine whether such a geometry may have any advantages in terms of a better cooling effect and a more effective removal of the preparation material due to the increased water supply. The SEM images showed undisturbed diamonds, and small amounts of debris were present in the spiral. However, the spiral apparently remained almost free of abraded particles. In contrast to conventional instruments, the special instruments (ABACUS) are diamond-coated using a special process with one matrix (UniMatrix) instead of several matrix steps for conventional systems. The abacus systems have a special layer applied after diamond coating, which is intended to enable the instrument to last longer due to the higher overall hardness. With this technology, the grinder is expected to have a higher diamond density than conventional diamond drills. Further on, this process is supposed to even the grain distribution and define the chip spaces for all diamond grinders. The SEM pictures partly confirm this, showing fewer exposed diamonds in comparison to the conventional coating systems.

### 4.2. Zirconia

On zirconia, both conventional instruments showed a clear loss in weight removal of about 25% after the fourth cut, which was only significant for one grinder. Conventional diamond burs, which are not specifically marketed for cutting zirconia, sometimes prove to be just as efficient as special diamond burs [17,18]. The weight removal with the first cut was approximately 20 mg and thus clearly higher in comparison to that of the instrument with the special diamonds with approximately half the weight removal. The results confirm the good cutting performance of diamond on zirconia [15]. In contrast, Gonzaga et al. found that the cutting efficiency did not decrease as the number of cuts increased [19]. The SEM images showed clear wear and erosion of the diamonds in the standard systems. Interestingly, however, the removal level for the special grinder remained at the same low level even after four applications. Together with the SEM images, this could be an indication of the good integrity and stability of the special diamond size and nickel coating of the ZS16 system. In contrast to the conventional systems, the structure of the diamonds was completely retained. If the weight removal of this system could be improved, it might be a good option for the sustainable machining of zirconia. The special bond of the Z-Cut instruments in combination with harder diamond grit is supposed to match to the extreme strength of zirconia ceramic to extend service life and improve cutting efficiency. The special character of the diamonds is confirmed by the SEM images, showing a clearly more geometric form of the diamonds. In addition to the special diamonds, all the other diamonds showed clear abrasion due to the cutting steps. Diamond grit fracture was the most dominant wear pattern. As a result of the diamond treatment, the zirconia surfaces were expected to show plastic deformation as evidence of ductile cutting [18]. Zirconia blocks, which were machined with fine grit diamond instruments, showed the least incidence of surface flaws. Consequently, fine-grain diamond instruments showed the lowest number of surface defects [18]. Fine-grit instruments (between 40 and 50 µm) were shown to be most efficient, achieving a high cutting depth without macroscopic damage to the zirconia [20].

The limitations of this study are certainly that only the weight loss criterion was determined. Although it was possible to differentiate between the different amounts of weight removal, the overall level was low. Interestingly, the level of weight loss for the weaker composites was only slightly higher (up to 20%) than that for the significantly harder and stronger zirconia. This could be due to the uniform geometry of the grinders or to a comparable size and arrangement of the diamonds on the surface of the grinder. It was also interesting that the weight loss after four cuts on the zirconia and composite was at a similar level. However, the different grinding times of 40 s (composite) and 5 min (zirconia) must be taken into account. As expected, the processing of zirconia takes considerably longer due to the different material properties [21]. It was shown that hardness, E-modulus, and flexural strength have an influence on grinding performance. Zirconia had to be ground five times longer to achieve the same weight removal as for the resin-based composite. 

## 5. Conclusions

The grinders used on resin-based composite and zirconia provided significantly different initial wear removal and durability. The grinders had comparable durability on the zirconia and resin-based composite. Grinding on zirconia took five times longer to achieve comparable weight removal.

## Figures and Tables

**Table 1:** Instruments for resin-based composite and zirconia treatment (*: NTI-Kahla; D; °: all Komet Dental, D#; all grinders: l = 8 mm; speed for all: 200,000 rpm).

	Material	Diameter [mm]	Type/Diameter		Comment	Comment	Composition/Properties
Resin-based Composite	Grandio blocs 14L A3.5 HT; VOCO, D	1.6	Z881-016C-FG “ABACUS” *	CS16	Abacus diamond, special coating	8 cuts/40 s per cut	Urethandimethcrylate, dimethycrylate, 86 wt. % filler, flexural strength 220 MPa, E-modulus 18 GPa, Vickers HV 122
			881-016C-FG *	CA16	Diamond, galvanic bond		
			6881.314.016 °	CB16			
		1.4	881-014C-FG *	CA14	Diamond, galvanic bond		
			6881.314.014 °	CB14			
			881-014TC-FG “TURBO” *	CS14	Diamond, spiral form		
Zirconia 3Y-TZP	Cercon base 30 colored; DeguDent, D	1.6	K881-016M-FG “Z-CUT” *	ZS16	Special diamond/bond	4 cuts/5 min per cut	Yttriumoxide 5%, hafniumoxide <3%, aluminiumoxide, siliziumoxide, oxide <2%, flexural strength 1200 MPa, E-modulus 210 GPa, Vickers HV1 380
			881-016M-FG *	ZA16	Diamond, galvanic bond		
			881.314.016 °	ZB16			

Scanning electron microscopy (SEM, Phenom, FEI; magnification 500×, working distance ~480 µm) of the instrument surfaces were taken before and after testing. The resulting surfaces on the specimens were also imaged by scanning electron microscopy.

**Table 2:** Weight loss [mg] for resin-based composite (mean, standard deviation, statistical comparison ANOVA, Bonferroni, α = 0.05).

	Mean	Std	Mean	Std	1st Significant Step to 1st Cut	Bonferoni Comparison (Cut 1:Cut 8)
	1st cut		8th cut			*p*
CS16	0.21	0.03	0.13	0.05	4	<0.001
CA16	0.25	0.05	0.16	0.05	7	0.023
CB16	0.21	0.06	0.09	0.05	6	0.001
CS14	0.24	0.03	0.12	0.06	3	<0.001
CA14	0.23	0.03	0.13	0.05	4	<0.001
CB14	0.19	0.02	0.11	0.02	4	<0.001
*p*	0.009		0.049			

**Table 3:** Weight loss [mg] for zirconia (mean, standard deviation, statistical comparison ANOVA, Bonferroni, α = 0.05).

	Mean	Std	Mean	Std	1st Significant Step to 1st Cut	Bonferroni
	1st cut		4th cut			*p*
ZS16	0.08	0.02	0.07	0.01	--	1.000
ZA16	0.21	0.05	0.15	0.04	--	0.056
ZB16	0.20	0.02	0.15	0.04	4	0.006
	*p* < 0.001		*p* < 0.001			

## Data Availability

The authors state that they did not use any external data.
